# The Pandemic beyond the Pandemic: A Scoping Review on the Social Relationships between COVID-19 and Antimicrobial Resistance

**DOI:** 10.3390/ijerph18168766

**Published:** 2021-08-19

**Authors:** Luisa Toro-Alzate, Karlijn Hofstraat, Daniel H. de Vries

**Affiliations:** 1 Amsterdam Institute for Global Health and Development, Paasheuvelweg 25, 1105 BP Amsterdam, The Netherlands; hofstraat@aighd.org (K.H.); d.h.devries@uva.nl (D.H.d.V.); 2 Department of Anthropology, University of Amsterdam, Nieuwe Achtergracht 166, 1018 WB Amsterdam, The Netherlands

**Keywords:** antimicrobial resistance, COVID-19, social sciences, social dimensions

## Abstract

The social sciences are essential to include in the fight against both public health challenges of antimicrobial resistance (AMR) and COVID-19. In this scoping review, we document what social science knowledge has been published about the social relationship between COVID-19 and AMR and which social science interventions are suggested to address this social relationship. We analysed 23 peer-reviewed articles published between 2019 and 2021. Results emphasize that changes in antibiotic prescription behaviour, misinformation, over-burdened health systems, financial hardship, environmental impact and gaps in governance might increase the improper access and use of antibiotics during the COVID-19 pandemic, increasing AMR. The identified social sciences transformation strategies include social engagement and sensitisation, misinformation control, health systems strengthening, improved infection prevention and control measures, environmental protection, and better antimicrobial stewardship and infectious diseases governance. The review emphasizes the importance of interdisciplinary research in addressing both AMR and COVID-19.

## 1. Introduction

Antimicrobial resistance (AMR) and COVID-19 have many similarities from a public health perspective. Both have a significant demographic and economic impact, both have a lack of medical treatment options and require behavioural changes to prevent their spread, and both need to be addressed through an interdisciplinary approach [1].

Although more and more is known about the relationship between COVID-19 and AMR, most information is biomedical. Social sciences research on AMR remains low [2], even though social sciences are a crucial element to face complex public health challenges such as AMR and COVID-19 from an interdisciplinary perspective. In this scoping review [3], we document what social science knowledge has been published about the social relationship between COVID-19 and AMR, what actions have been suggested and generated to deal with the social dimensions of AMR and COVID-19 [4], and we explore further the role of social sciences as a transformation agent.

**Figure 1:**
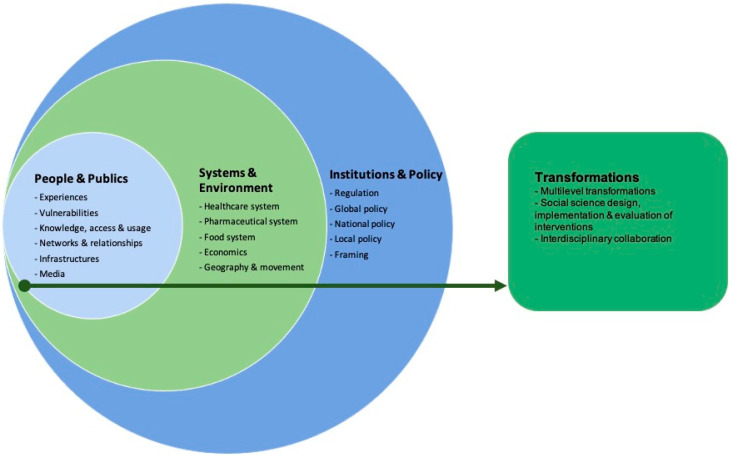
SPECIAL SOC AMR Framework.

We define the social dimensions of AMR as the scalar relationship between people (their knowledge, behaviours, experiences and social networks), systems (e.g., the health system), the environment (including economics, geography) and policy-making and how they are affected by AMR, and vice versa [4]. The analytical framework (Figure 1) was developed during an expert meeting in 2019, organised through a three-year EU-funded project on integrating social sciences in epidemic threats (www.sonar-global.eu, accessed on 15 August 2021). In the meeting, we discussed the social dimensions of AMR to generate a curriculum for trainers (SPECIAL SOC AMR) [4]. Thus, the curriculum aims to guide trainers on how to instruct social scientists on the social dimensions of AMR from the biomedical scale to the global governance scale. In this case, the curriculum framework was adapted to specifically look at the social dimensions, omitting the biomedical aspects [4].

The framework encompasses four main scales, complemented by an action-oriented focus on social science-based transformations:

- People and publics: portraying prescription practices, knowledge about antibiotics, behavioural practices of consumption and access to antibiotics, its impact on vulnerable groups, the social networks and relationships (including user-prescriber relationship), and the role of media on the prescribing and consumption of antibiotics during the pandemic.
- Systems and environments: framing the dynamics and interactions between AMR and the healthcare, pharmaceutical and food systems. It also includes the economic impact and the role of geography and movement on AMR.
- Institutions and policies: exploring the AMR policy-making from the local (hospital stewardship) to the national and the global level.
- Transformations: integrating the mitigation strategies advised and developed to tackle AMR during pandemic times, the social sciences’ role, and the resulting interventions. These transformations also cover the collaboration between social scientists and non-social scientists.

We searched for more recent studies regarding AMR and social sciences to adapt the framework to recent developments within the field. Two recent frameworks were found which address the social dimensions of AMR [5,6]; they, however, did not identify any additional social dimensions or elements that could be added to our current framework. 

Using this framework, we have answered the following main questions:

1. What evidence is available in peer-reviewed research that addresses the social dimensions of the relationship between AMR and COVID-19?
2. What actions are suggested and generated to deal with these social dimensions?
3. What is the role of the social sciences as a transformation agent during the COVID-19 pandemic?

## 2. Materials and Methods

### 2.1. Search Strategy

This review included papers published in peer-review journals between December 2019 and February 2021 in English and Spanish languages. This scoping review followed a methodological framework for scoping studies [3]. Because it was seen as a scoping review, registration of the review protocol was not necessary.

We searched index terms and selected keywords in MEDLINE, JStor and Google Scholar in February 2021. We limited all searches to full-text articles published in English and Spanish between 2019 and 2021. The search strategy included the following search terms: (“Antimicrobial Resistan*” OR “Anti-microbial Resistan*” OR “Antibiotic Resistan*” OR “Drug Resistan*”) AND (“Social Scienc*” OR “Health Econom*” OR “Management stud*” OR “Business stud*” OR Education OR Anthropolog* OR Linguistics OR Law OR Histor* OR Politic* OR “International relations” OR Psycholog* OR Sociolog* OR “Science and technology stud*” OR “Communication science*” OR “Cultural Stud*”) AND (“COVID 19” OR “SARS-CoV 2”). These keywords were selected to make sure to find articles that reflected on both antimicrobial resistance and COVID-19, while using a social sciences perspective.

### 2.2. Inclusion and Exclusion Criteria

In line with the pre-set research questions in advance of the data extraction process, we determined a set of inclusion and exclusion criteria. These included:

1. The article addresses both topics (AMR and COVID-19) throughout all its content;
2. The article is published in a peer-review journal, including commentaries, review articles, editorials, and viewpoints;
3. The content of the article includes a social sciences perspective.

### 2.3. Study Selection

**Figure 2:**
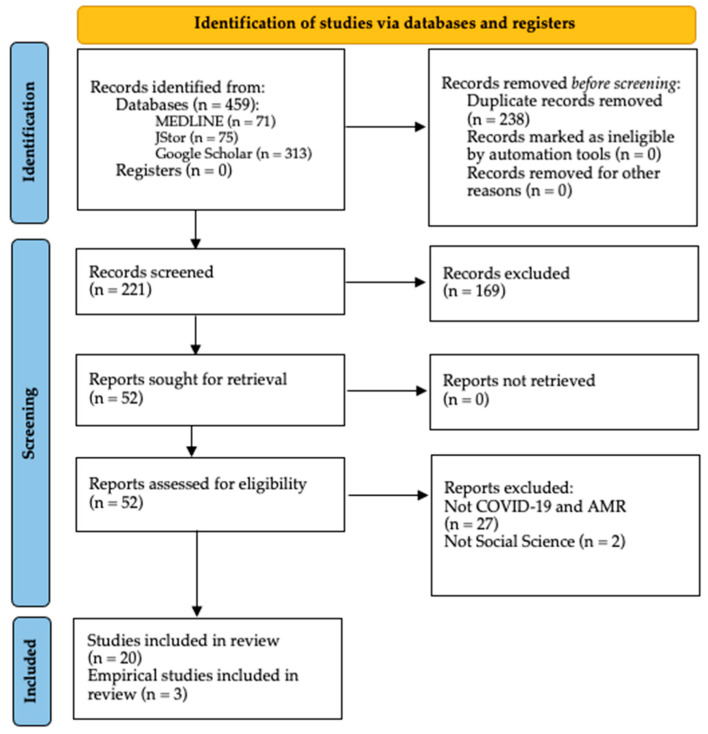
PRISMA flow diagram.

The total number of articles found during the initial search was 459 (see Figure 2). After removing the duplicates, a screening was applied to the titles and abstracts, retrieving 52 articles for full-text eligibility. In this screening process articles were only selected if their titles and/or abstracts reflected that their primary focus was AMR and COVID-19, as well as a relationship with at least one social science. Twenty-three articles were selected for analysis after applying the selection criteria. All the items included in this review were published in peer-review journals, and most of the articles (*n* = 20) were published expert opinions, viewpoints, commentaries or editorials. The remaining three (*n* = 3) presented empirical data, including qualitative methods, questionaries and time-series analysis.

All the information about the articles, including name, author, study characteristics and other relevant information following the analytical framework, were extracted using an Excel-based data extraction sheet, coded for all the framework categories. First, the data extraction process was calibrated by all the authors (LT, DDV and KH) based on a small sample. Then one author (LT) proceeded to extract the information and conduct the preliminary aggregation of results discussed by the team at several stages.

## 3. Results

This section provides all the detailed findings of this scoping review. A summary with the key findings can be seen in Table 1.

### 3.1. People and Publics

#### 3.1.1. Antibiotic Prescription Behaviour, Knowledge and Access

Literature reports great concern among healthcare workers regarding excessive prescription rates, the use of broad-spectrum antibiotics, prolonged antibiotic treatments without clinical justification, and inappropriate prescribing of antibiotics to patients infected with COVID-19 [7,8,9,10,11,12]. For example, in the United Kingdom, Zhu et al. found that clinicians prescribed more broad-spectrum antibiotics due to the inability to assess patients face-to-face [13]. In Spain, Abelenda-Alonso et al. found that before the pandemic, the antibiotic consumption in January and February 2019, compared with the same months in 2020, was similar. However, during March and April 2020, the antibiotic consumption increased significantly compared to the same months in 2019, probably because of the lack of knowledge about the infection and the lack of formal stewardship programmes in the emergency response [14].

While consultations via telehealth were used as a tool to treat patients and avoid their presence in the healthcare facilities and the possible spread of COVID-19 infection, its use may have increased antibiotic prescription due to the unavailability of syndromic diagnostic panels, and the challenge for clinicians to determine the nature of the illness, resulting in a potential increase of inappropriate prescriptions of antibiotics [15]. In addition, the eagerness to fulfil the patient’s perceived expectations regarding COVID-19 infection and treatment could increase antibiotic prescription by healthcare workers [16]. Although these elements existed previous to the pandemic, it is clear that the emergence of COVID-19 and the uncertainty it created on a global scale has fit in this same pattern and most likely exacerbated it.

The COVID-19 challenges combine with ongoing lack of knowledge about proper antibiotic use among health professionals, including a “just-in-case” prescription [8,17,18], and the use of experimental antibiotic treatments due to the absence of specific treatments against COVID-19 that might increase the use of non-essential antibiotics [9,11,17]. The “just-in-case” prescription was related to the decrease in education and training activities included in the AMS programmes, negatively affected by the pandemic [8]. Added to this is clinical uncertainty about COVID-19, leading clinicians to prescribe antibiotics without medical indication, based on the perception that the potential benefit was greater than the risk [16,17].

Due to postponed healthcare-seeking behaviour during COVID-19 pandemic, antibiotic utilisation increased in response to the delayed presentations of acute infectious conditions, resulting in the prescription of antibiotics used for more severe and complex infections without bacterial confirmation [16]. In addition, some authors speculated that there is likely also an increase in self-medicating behaviour, especially in communities where antibiotics are easily accessible without a prescription, either as a preventive or as an empiric measure against COVID-19 or non-COVID infections, as people avoid public consultation at healthcare facilities [10,19,20,21,22]. In Iran, for example, at least 20% of study participants were consuming self-prescribed antibiotics because of the fear of leaving home to find medical treatment for their illness, and wanting to avoid overcrowded places, hospitals and clinics [20].

#### 3.1.2. Vulnerable Populations

An increase in AMR strains is anticipated particularly in low-and-middle-income countries (LMIC) as a result of the disruption of antimicrobial stewardship programmes, limitations on laboratory capacity, and deficient regulation on antibiotic access [15]. In other words, COVID-19 unequally affects more vulnerable populations already forcibly living in less hygienic and safe circumstances with a higher probability of environmental spread of resistant bacteria. Furthermore, in many LMIC, the re-allocation of resources to respond to the new demands in the health system tends to impact the control of MDR tuberculosis disproportionally, adding more burden to the already more fragile health systems [15]. Mobile populations with a high prevalence of resistant bacterial strains and COVID-19 infections, like travellers, migrants and refugees, have also been identified as being at higher risk of complications from COVID-19 secondary infections [15]. This risk of secondary infections and increased use of antibiotics due to health system disruptions during the COVID-19 pandemic can also be seen in cancer and multi-drug-resistant tuberculosis patients [18].

#### 3.1.3. Social Relationships and Networks

Some AMR “positive” outcomes could be seen from the change in the social interaction between healthcare services and people. First, due to the disruption of many routine health services, patients are not receiving elective care, nor are antibiotics prescribed, overall reducing the AMR exposure [9]. Second, the behavioural interventions to prevent the spread of COVID-19 infection, which includes physical barriers and social distancing, likely also mitigate the increase of other kinds of infections, including bacterial, diminishing, as a consequence, antimicrobial use [9]. In addition, due to self-isolation and the avoidance of the health services because of the lockdowns and the fear of COVID-19 exposure, there was a decreased demand for necessary antimicrobials and on antibiotic prescription in emergency rooms and day clinics [13,15,16]. On the negative side, impacted by overcrowded health services and overburdened clinicians, consultation time is minimal due to the increase in patient numbers, negatively affecting clinician-patient communication, impairing the information and education that the patients receive on proper antibiotic usage [18].

#### 3.1.4. Media and Misinformation

Misinformation has been a primary driver in the misuse of antibiotics during the COVID-19 pandemic. In many countries, misinformation included a widespread emphasis by political leaders and media on the potential role of antimicrobials in the prevention and treatment of COVID-19 infection. A notable example was the suggested use of chloroquine (an antimalarial drug) plus azithromycin (an antibiotic used in respiratory bacterial infections) to treat COVID-19 disease. The initial media coverage of the COVID-19 outbreak highlighted that there was no “cure” or “treatment” for infection [23]. Later on, some media reports and political leaders magnified using azithromycin together with chloroquine as a treatment for COVID-19 infection [17]. A study with several methodological limitations and high media exposure originated this practice, which increased the consumption of azithromycin [19,20] and other types of antibiotics not only among medical professionals but also among the general population [15]. One expert from Kaiser Permanente (Washington DC) illustrates the prevailing concern: “If we keep having more inflammatory claims about antibiotics that may help, like azithromycin, then we are going to have patients who come in demanding it as soon as they hear they have COVID-19” [17] (p. 2).

In many countries, the politicisation and underestimation of the pandemic by public health stakeholders have turned politicians into a leading source of misinformation, increasing beliefs in conspiracy theories. Widespread misinformation and inconsistency of information have led to public confusion [10]. For example, in the United States, initial estimates reported at least 32,000 prescriptions of azithromycin in the days after the President declared this antibiotic could cure COVID-19 infection [10].

Another issue identified is the early posting of preprints which has allowed the fast spread of scientific information of questionable quality, and, with this, the dissemination of inaccurate information among the general population [10,23]. Furthermore, this inaccuracy in the information, the low awareness of the correct use of antibiotics and the lack of basic knowledge about the origin of the infection had brought a poor understanding of the pandemic and its origins among the general population worldwide [15].

### 3.2. Systems and Environments

#### 3.2.1. Healthcare System

The COVID-19 pandemic has had a huge impact on all health systems worldwide. The disruption in routine services such as immunisation, antibiotic treatment, laboratory services, and infectious diseases control and surveillance programmes (as for multi-drug-resistant tuberculosis and HIV) increases the misuse of antibiotics [15,24]. Disarrays in immunisation services may bring critical long-term consequences, such as outbreaks and re-emergence of vaccine-preventable diseases, increased risk of infections and, as a consequence, overuse of antibiotics [17,25]. In addition, the increase in hospital admissions due to COVID-19 infections could raise the risk of healthcare-associated infections and the spread of multidrug-resistant bacteria, exacerbating antibiotic use [15].

The impact of the COVID-19 pandemic on multi-drug-resistant (MDR) tuberculosis and HIV treatment and control may be seen as a result of the increase in the demands of health resources [10,24]. The interruption of HIV and TB treatment may increase drug resistance, together with the disruption in access to laboratory and follow-up services, increase the risk of opportunistic infections and the emergence of new resistant strains of these infections [11,22]. Data from WHO compilated in 84 countries during 2020 shows a reduction of 21% of people receiving care for tuberculosis, approximately 1.4 million fewer people than in 2019, related to COVID-19 disruptions in access to TB care [26].

The shortages in the health workforce and the limited laboratory capacity induced by the pandemic may delay the diagnosis of other infectious diseases and decrease the capacity to detect AMR microbes, elevating antibiotic use as a consequence [15]. Furthermore, the high need for supplies like personal protective equipment (PPE) for patients with COVID-19 may reduce resource availability to prevent and treat other infectious diseases [11]. This high need, together with the shortages in PPE, the low adherence to standard infection prevention and control practices (IPC) by healthcare workers and the overcrowded healthcare facilities, likely increase the transmission of AMR bacteria and antimicrobial use [27].

#### 3.2.2. Pharmaceutical System

For the last 30 years, the lack of development of new antibiotics—referred to as a “discovery void”—has been due to the paucity of research on antimicrobials [10,28,29]. Due to the COVID-19 pandemic, the need to discover a treatment has further reassigned resources allocated to develop new vaccines, new antibiotics, and therapeutic application projects [10]. Furthermore, the development of clinical trials for antibiotics might be disrupted by the pandemic as the research facilities and hospitals try to address the needs of COVID-19 patients, and as priorities for the research agenda changes [9]. Additionally, in order to identify the most effective ways to treat and improve outcomes in COVID-19 patients, several antimicrobials must be tested in clinical trials or ad hoc studies, with the risk of exacerbating AMR rates [10].

The disruption produced by the COVID-19 pandemic may affect the production chains in several industrial processes, including production, distribution and delivery of vaccines and antimicrobials [11]. Furthermore, the shortages and redirection of funding could affect small and medium-size production laboratories, which usually produce vaccines and antibiotics destined for local markets [25]. This possible shortage of narrow-spectrum antibiotics and vaccines might increase AMR [10,21], raising the use of broad-spectrum antibiotics used to treat severe infections and the spread of vaccine-preventable diseases.

#### 3.2.3. Economics

The global supply chains of antimicrobials and vaccines tend to be affected by the pandemic in several ways, including an increase of the cost due to travel restrictions and disruptions in trade between countries, the shortages of the workforce involved in the production of these vaccines (because of illness or relocation) and the development of protectionist policies (including the relocation of funding for COVID-19 supplies) [21]. This disruption in the supply chain is likely to be especially challenging in countries with high dependence on imported medicines and pharmaceutical supplies, putting patients’ lives at risk and contributing to drug resistance, as many antibiotics and vaccines become unavailable [10]. The pandemic might affect antimicrobial and vaccine production as a consequence of shortage of external funding for local laboratories. As the production stops, revenue streams may cease, threatening the operation and the survival of these companies, with the risk to close permanently without external financial aid [25]. Some authors suggest another economic impact might be on agricultural activities considering the spread of COVID-19 infection into rural areas, as COVID-19 also affects the agriculture workforce, which could potentially leave animals and crops unattended [10,25]. The authors suggest this might stimulate the use of antibiotics in animal production as a desperate measure trying to maintain the market demand, using antibiotics as prevention of infections in animals living in overcrowded conditions [24].

#### 3.2.4. Environment

Strict hygiene and sanitation measures are one of the cornerstones in the prevention of COVID-19. Due to this, the use of biocidal agents for environmental and personal disinfection in healthcare and non-healthcare settings could contribute to the natural selection of drug-resistant bacteria, as these are now increasingly exposed to low levels of biocidal agents [23,30]. In addition, both biocides and antibiotics used in the treatment and prevention of secondary bacterial infections in COVID-19 patients are likely to be detected in wastewater treatments plants, rivers and coastal waters, increasing levels of AMR in the environments and putting the individuals exposed at risk [19].

Unmeasured until now, the potentially high consumption of antibiotics from prescriptions and self-medication by the general population during the pandemic may lead to a high proportion of bioactive forms being excreted into the wastewater, from where they may enter into natural systems. This consequence might be more prevalent in countries where antibiotics can be easily obtained without a prescription [19]. The presence of high levels of biocides in wastewaters, and the use of wastewater biosolids as soil conditioners with higher concentrations of antibiotics, may create a threat to the ecosystem and the spread of AMR in the environment. Moreover, these biocides in the environment can disrupt the wastewater treatment process that depends on microbial activity. The presence of these compounds in the soil system can threaten the functionating of native microbiota and its role in biogeochemical cycles [19].

### 3.3. Institutions and Policies

#### 3.3.1. Local Policies (Hospital Stewardship Programmes)

Several antimicrobial stewardship (AMS) programmes were established after releasing the Global Action Plan for Antimicrobial Resistance. Unfortunately, after the beginning of the COVID-19 pandemic, many of these programmes were reduced in terms of both workforce and stewardship activities (audit, quality assessment, education and training, improvement initiatives, hospital rounds), as COVID-19 was prioritised above AMR [7,8,10,12,17,24]. Hospitals made sure they had sufficient supply of antibiotics, however, the limited resources for antimicrobial stewardship programmes indirectly impacted antibiotic use, leading to an increase in AMR [17]. The insufficient data available makes it harder to predict the impact this pandemic will have on antimicrobial stewardship programmes and, subsequently, in long-term rates of AMR [12].

In African countries, where the scarcity of resources allowed only a few countries to have antimicrobial stewardship programmes before the pandemic, all the resources for AMS-related activities were shifted towards the pandemic response [31]. According to Chibabhai et al., in South Africa, there are not enough infectious disease physicians to cover the demand of AMS services as they are now in the frontline against COVID-19, severely impacting AMS activities in hospitals. In addition, the scarcity of pharmacists in most public sector hospitals did not permit any of them to be exclusively dedicated to AMS activities, even before the pandemic [25].

Surveillance data are likely to be inaccurate due to the unreported amounts of antimicrobials (antibiotics and antifungals) administrated worldwide during the first moths of pandemic [32]. As experts fear the slowing down of the global initiatives to face AMR, many of them emphasise the need for data collection on how AMR may be affected by the healthcare response to the pandemic. For example, the US Department of Defense is researching the rates of secondary infections and antibiotic use in patients with COVID-19 infection [17].

#### 3.3.2. National and Global Policies

Antimicrobial resistance legislation showed delays for nationwide implementation, despite the call of the WHO Global Action Plan for Antimicrobial Resistance for an effective and fast implementation phase of the National Action Plans. In this way, due to the pandemic, many national plans and other initiatives against AMR are likely to be delayed, temporarily ceased or postponed [11]. These delays and cessations can be seen, for example, in many African countries [31], likely increasing AMR.

### 3.4. Transformations

The papers reviewed several social sciences strategies to tackle AMR during the COVID-19 pandemic. For many of the outlined issues, a synergy seems to be achievable while directing attention to the most critical roles social sciences can play in dealing with AMR in the context of the COVID-19 pandemic. A comprehensive overview of all suggested strategies is shown in the Table A1. Below we will summarize the main suggestions in the papers reviewed.

#### 3.4.1. Social Engagement and Sensitisation

Fortunately, new measures implemented to decrease the spread of COVID-19 infections, such as hand washing, physical distancing and quarantining, became part of people’s everyday routine and can also be effective to reduce AMR infections and the spread of AMR microbes [9,28]. In addition, spreading awareness about proper antimicrobial use and educating the general population about the lack of evidence for antibiotics as a proper treatment for COVID-19 can also decrease improper antibiotic use [21,31,33]. Furthermore, patient education on the correct use of antibiotics can play a role as a transformation strategy, engaging the community on the proper use of antimicrobials [21].

#### 3.4.2. Misinformation Control

With increasing awareness, the control of misinformation should be a priority, together with the generation of reliable and trustworthy information about COVID-19 and antimicrobial use, accessible for both healthcare workers and the general population [7,18]. For this case, health communication strategies should be developed to assure that trustworthy information is spread by politicians and public figures, therefore directly impacting the general public.

#### 3.4.3. Health Systems Strengthening

Another element highlighted is the strengthening of health systems needed to resist the pandemic’s impact. Investment in expanded capacity building of hospitals and laboratories to face the growing number of patients and the improvement of testing methods for COVID-19 to reduce antibiotic prescription will also help reduce the possibility of increased AMR [24,28]. Further, the continuity of essential services, including antibiotic supply and vaccines, can prevent increasing numbers of AMR cases developed due to COVID-19 pandemic [24]. Capacity building to strengthen the health systems turns out to be an essential tool to decrease both COVID-19 and AMR infections. The capacity building should be focused on adequate training of health workers on COVID-19 and AMR, expansion of high-quality virtual consultations systems with trained personnel to reduce self-medication practices during lockdowns and avoiding overcrowded hospitals, and the development of digital platforms to communicate accurate information about COVID-19 and the ineffectiveness of antimicrobials as treatment [7,18,20].

#### 3.4.4. Infection Prevention and Control

The application of infection prevention and control (IPC) measures learned during the COVID-19 experience might be helpful as leverage to improve IPC measures and decrease AMR [28]. The compliance and correct application of these IPC measures is essential to control the spread of both COVID-19 infection and AMR bacteria [11].

#### 3.4.5. Environmental Protection

The use of biocides for disinfection to diminish the spread of COVID-19 has become one of the most important measures against the virus. To diminish the environmental impact that biocide products can have, community and healthcare institutions must be cautious with the amount and type of biocides used, as well as their disposal methods [24].

#### 3.4.6. Antimicrobial Stewardship

Concerning antimicrobial stewardship programmes, it is crucial to evaluate the impact of the COVID-19 pandemic on antimicrobial use and to strengthen and prioritise the routine AMS programmes in order to prevent the inappropriate prescribing of antimicrobials in COVID-19 patients [15,19,28]. Furthermore, antimicrobial use should be aligned with recommendations released by the WHO on the use of antimicrobials in COVID-19 patients and clear protocols should be provided to healthcare workers [21,31]. While the cost of implementing AMS programmes might be an obstacle during the pandemic, savings due to the reduction of drug costs, length of hospital stays and readmission rates, can provide leverage and incentive for implementing proper AMS programmes despite their initial high implementation cost [33].

#### 3.4.7. AMR and Infectious Disease Governance

The establishment of a strengthened governance framework with national policies assuring essential public health programmes, improved collaboration between international health agencies and national governments, and transparent reporting and surveillance of AMR during and after COVID-19 times, can help to continue the fight against AMR despite the impact of the pandemic [10,19,21,28].

## 4. Discussion

Overall, the published articles and commentaries included in our scoping review showed a relative biomedical emphasis without much in-depth consideration of how the pandemic affects other spheres, such as social, cultural, economic, environmental and governance dimensions. Details on prescribing practices, health systems challenges, and stewardship programs from the perspective of health care workers are dominant in the articles reviewed. Two other recently published reviews on the same topic confirm this sentiment. A review by Knight et al. analysed how AMR emergence, transmission, and burden is affected by COVID-19 in terms of antimicrobial usage, infection prevention, and health system functioning [34]. Rodriguez-Baño et al. addressed the weaknesses regarding appropriate antibiotic use during the COVID-19 pandemic, with the risk of increasing AMR, due to the low priority given to AMS and AMR surveillance programmes [35]. Although these two reviews share several perspectives with our review in relation to antimicrobial use, health-seeking behaviour, impact on the health system and the need for strategies against the increase of AMR due to COVID-19 infection, their general perspectives do not take into account social insights and relationships, such as the role of culture, people’s perspectives, governance and the consequent need for interdisciplinary collaboration. Aside from the overall lack of evidence and speculation in the reviewed articles, the dominance of biomedical studies gives little space for interdisciplinary collaborations, and fails to reduce the gap between social and biomedical sciences.

Focusing on the social sphere, we found that the pandemic brought changes in antibiotic prescription practices, antibiotic consumption and health-seeking behaviour. These findings are compatible with the reviewed findings of Knight et al., in so far as they describe how inappropriate prescribing and misdiagnosis, lack of another treatment, and the “desire to try all avenues” has led to the continuing prescription of antibiotics to treat COVID-19 infections by health professionals [34].

In addition, we found that telemedicine might increase the over-prescription of antibiotics due to sub-optimal decision making by physicians. Knight et al. also refer to how telemedicine is not compatible with many settings globally, increasing the over-prescription of antibiotics [34]. One study carried out before the pandemic showed how education and individualized feedback on antibiotic use for clinicians could improve antibiotics prescribing for upper respiratory infections via telemedicine consultations [36]. This highlights that if the right measures in terms of antimicrobial stewardship implementation and commitment of governments and healthcare institutions to guarantee access and quality of services are taken [37], telemedicine could be used as a backup measure in pandemic times without increasing antibiotic prescription rates.

Another social science concern addressed in our scoping review is the impact on vulnerable populations, such as migrants and people living in LMIC during the pandemic. These populations may have a lack of access to antibiotics and infection prevention measures, as well as a higher prevalence of AMR strains in mobile populations and a higher risk of complications due to COVID-19 secondary infections. However, it remains unclear how the pandemic can affect the incidence of AMR in Black, Asian, and minority ethnic (BAME) groups. As studies have shown, socially marginalised populations have been worse affected by COVID-19 [38,39,40,41]. During the SARS pandemic in 2003 the Asian-American community was affected by stigmatisation and discrimination due to the term “Chinese virus”, and this delayed care seeking and made the control of the virus outbreak harder, having social and economic consequences [42].

The results also show the high impact of low public awareness on AMR and misinformation around antibiotic use as a preventive measure or treatment for COVID-19, which may have increased the consumption of antibiotics among the general population worldwide. From the HIV pandemic, we have learned how misinformation can lead to mistrust of governmental initiatives, and also how social media facilitates the spread of conspiracy theories creating a state of social paranoia [43]. In the COVID-19 pandemic, we see again how misinformation is filling the knowledge gap about the infection, increasing misuse of antibiotics, leading to the development of conspiracy theories and diminishing the adherence to behavioural recommendations. Therefore, social science insights are needed again to address this issue that remains under-researched.

Our findings agree with the review by Knight et al. regarding the economic and environmental dimensions. With respect to economics, the impact of the COVID-19 pandemic on AMR was seen in the increased cost of antimicrobials, the shortages in the workforce and the development of protectionist policies affecting mainly LMICs. Knight et al. describe how financial hardship may affect access to antimicrobials and increase AMR infections and mortality [34], findings compatible with the outcomes of this review. Environmental effects were addressed as well by Knight et al., noting the possible impact of the food system increasing AMR prevalence within the animal-based food supply chain [34], a finding not addressed by the articles in our review. This indicates that the One Health nature of AMR and the role of the COVID-19 pandemic needs to be prioritised in the AMR research field.

National and global governance on AMR has been affected by the pandemic by delaying or ceasing the development and implementation of plans and programmes. In its review, Rodriguez-Baño et al. (2021) addressed the difficulty of enforcing regulations on antibiotic use, especially in settings where access to antibiotics without prescription is easy, and counterfeit medicines are openly available [35]. In addition, we saw how the Ebola crisis in 2014 exposed several governance gaps in infectious diseases control, due mainly to the late response of the World Health Organization (WHO) and other international agencies. This led to a high death toll and the impairment of many national health systems [44]. However, none of the reviews used to compare our results addressed the topic of governance of AMR during pandemic times, showing a new gap in AMR research and the urgent need for policies and programmes addressing AMR-both now and after the time of the COVID-19 pandemic.

The transformative social science strategies we found in the reviewed papers aim to decrease the spread of COVID-19 infection and its impact on AMR, and slow the pace of the foreseen consequences of AMR in all aspects. The strategies provided by the authors are mostly generic, lacking sophisticated social science analyses, and aligned with the mainly biomedical “mitigation strategies” and “key recommendations” suggested by Knight et al. and Rodriguez-Baño et al. [34,35]. This shows the need for an interdisciplinary approach, where biomedical strategies are complemented and reinforced by social, cultural and political measures.

Finally, when zooming in on antimicrobial stewardship (AMS), several studies underline that this service could and should be maintained during pandemic times through targeted online training, use of online guidelines, the development of antimicrobial management protocols and integrating AMS activities in the pandemic response [24,25,31]. 

## 5. Conclusions

Despite the many findings, we note a serious shortage of original research on the social relationship between COVID-19 and AMR. With only three exceptions, this review identified mainly commentaries and editorials which generally only skimmed the surface of this complex relationship and provided little evidence. The two existing review articles on the relationship between COVID-19 and AMR are dominantly focused on biomedical challenges. It is possible that this apparent lack of scholarly studies from the social sciences may be the result of the short history of COVID-19. However, this lack of evidence is not as noticeable in the overall social science attention to COVID-19 with many published papers. However, this state of affairs does echo the findings of Frid-Nielsen, Rubin and Baekkeskov (2019) that there is a dearth of social science scholarship on AMR. Combined with the observed decrease in training and education on AMR due to COVID-19, we believe this is serious cause for concern if we want to address these complex interactions during the current or future epidemics.

There is no shortage of future research topics addressing the social, economic, political and cultural dimensions based on the current findings alone: citizen or patient influences on provider treatments and prescriptions; self-medication; the influence on health seeking behaviour in the context of medical pluralism; the ambiguity of telemedicine; social impact on and lived experience of socially marginalised and vulnerable populations; the influence of misinformation, politicisation and even publication cultures on knowledge gaps; the impact of financial hardship on needed access to antibiotics; the need of policies addressing AMR governance during pandemics; etc. Once we look beyond our findings, even more areas of further research could be identified to look at the relationship between COVID-19 and AMR: the One Health interplay between the human, animal and environment in terms of relations, networks, systems and policies; governance issues, including political framing, policy transfer and practices (e.g., community engagement, agenda setting, blaming, power relations) as well as the role of social scientists in the design, implementation and evaluation of the mitigation strategies brought forward in this scoping review and the impact of social science methods and research behind them.

As for the practical implications of our findings, hospital managers should be aware of the need for development and better implementation of AMS policies and programmes, optimized telemedicine use and misinformation control even and maybe especially in times of COVID-19. This could improve the access to health services, availability, quality and affordability of health services, since adapting these services will result in a decrease in the need for and misuse of antimicrobials, and subsequently the healthcare costs even in overcrowded health services and high-pressure situations as during pandemic times. On the other hand, governments and international health agencies should strengthen and continue misinformation control on the lack of evidence of antibiotics used as treatment or prevention measure against COVID-19 as well as patient education on proper antimicrobial use. Moreover, they need to prepare and establish an action plan to prevent and deal with future pandemics that can affect and increase AMR worldwide.

We hope this scoping review raises the interest of many social sciences researchers to include AMR in their area and biomedical researchers to understand the non-biomedical side of AMR and COVID-19. It is positive to see that there is some overlap in strategies that reduce the AMR and COVID-19 burden, showing us that by addressing COVID-19 correctly, we can affect and decrease AMR as well. In that way, interdisciplinary work will allow us to face both pandemics from all fronts, making it plausible to overcome these two global threats.

## Figures and Tables

**Table 1:** Key findings of this scoping review following the suggested framework.

Framework Level	Framework Sublevel	Results
People and Publics	Antibiotic prescription behaviour, knowledge and access	Excessive AB prescription rates, use broad-spectrum AB, prolonged AB treatments, inappropriate antibiotic prescription Increase antibiotic use due to consultations via telehealth Eagerness to fulfil patient’s expectations and “just-in-case” AB prescription Lack of knowledge about proper use of antibiotics Decrease in education and training activities Clinical uncertainty Postponed healthcare-seeking behaviour Self-medication of AB practices
	Vulnerable populations	LMIC: Increase of AMR strains (low AMS services, laboratory capacity, deficient regulations) Impact on MDR-TB (resources re-allocation) Travellers, migrants and refugees: higher risk of COVID-19 complications
	Social relationships and networks	Impact in elective care and prescription of antibiotics Behavioural interventions mitigating the increase of other kind of infections Self-isolation and lockdowns: decrease demand for necessary antimicrobials Minimal consultations times, negatively affecting clinician-patient communication
	Media and misinformation	Political leaders and media coverage on potential role of AB for COVID-19 Politicians as leading source of misinformation Early posting of preprints: fast spread of questionable quality scientific information
Systems and Environments	Healthcare system	Disruption in routine services Disarrays in immunisation services Increased health-care associated infections Interruption of HIV and TB treatments Shortages in workforce Limited laboratory capacity High need of PPE Low adherence of standard IPC
	Pharmaceutical system	Re-allocation of resources for R&D pf antibiotics and vaccines No development of clinical trials for antibiotics Affectations of small and medium-size production laboratories Shortage of narrow-spectrum antibiotics
	Economics	Increase of the cost of antibiotics Shortage of antibiotics Impact in agricultural activities
	Environment	Natural selection of drug-resistant bacteria High antibiotic levels in wastewaters Change in microbiota
Institutions and Policies	Local, National and Global policies	Reduced AMS activities Inaccurate surveillance data of antimicrobial use National Action Plans for AMR delayed, temporarily ceased or postponed

## Data Availability

Not applicable.

## Appendix A

Table A1 illustrates the specific “mitigation strategies” suggested for all the review authors to deal with the increase of AMR during and after the COVID-19 pandemic.

**Table A1:** Mitigation strategies suggested by the reviewed authors.

Author	Social Engagement and Sensitisation (Awareness, Behaviour Change)	Misinformation Control	Health Systems Strengthening	Infection Prevention and Control Measures	Environmental Protection	AMR Surveillance and Antimicrobial Stewardship Programmes	AMR and Infectious Diseases Governance During COVID-19 Pandemic
Rawson, Ming, et al., 2020 [12]						Evaluate the impact of the COVID-19 pandemic on antimicrobial use, antimicrobial resistance and access to effective antimicrobial treatments. Keep and promote routine surveillance and AMS principles on AMR during COVID-19 times. Strengthen and prioritise antimicrobial stewardship programmes during pandemic times.	Review national policies that do not neglect essential public health programmes in TB and immunisation delivery.
Usman, Farooq and Hanna, 2020 [19]	Educate the public about the unwanted effects of antimicrobial/antibacterial products during the pandemic.					AMS should continue to be applied and promoted during COVID-19 times.	Development of an antimicrobial policy specific for COVID-19, with coordinated strategies at the individual, healthcare and policy levels.
Strathdee, Davies and Marcelin, 2020 [28]				Leverage infection control principles from COVID-19 experience to control AMR.		Prioritise antimicrobial stewardship programmes during the pandemic.	
Khor et al., 2020 [21]		Patient education on the appropriate use of antimicrobials and the lack of evidence that antibiotics can be used as a treatment for viral infections, including COVID-19.					Adherence to guidelines recommendations to prevent over- and inappropriate prescribing of antimicrobials during the pandemic.
Iwu et al., 2020 [31]						Integrate antimicrobial stewardship into the pandemic response will help to minimise the emergence of AMR during the pandemic. Local guidelines should incorporate the WHO guidance on the use of antimicrobials in the treatment of COVID-19.	
Getahun et al., 2020 [24]			Targeted training to increase clinical competence among health workers treating COVID-19 patients. Ensure the continuity of essential health services and regular supply of antimicrobials, including retroviral and tuberculosis drugs and vaccines.		Prioritise biocidal agents without or with a low selection pressure for AMR.		
Hsu, 2020 [17]						Collect data on how healthcare responses to the pandemic may be affecting AMR.	
Chibabhai et al., 2020 [25]						Development of COVID-19 management protocols by AMS teams Continue AMS activities in non-COVID-19 sections of healthcare facilities.	
Arshad et al., 2020 [18]		Development of digital platforms to correct antimicrobial misinformation showing the ineffectiveness of antimicrobials as a treatment for COVID-19 infection.					
Wilson et al., 2020 [7]			Prepare and strengthen health systems to the rising burden of AMR after pandemic by strength health systems through investments in capacity building, adequate training for healthcare personnel, adequate supply of antimicrobials and PPE.				
Miranda et al., 2020 [32]	Increase societal sensitisation towards infectious diseases and good sanitary practices during the pandemic to diminish the potential impact on rates and transmission of AMR.						
Nieuwlaat et al., 2020 [9]	The behavioural changes implemented to deal with the COVID-19 pandemic would also be beneficial in dealing with AMR, as both face similar paths.						
Yam, 2020 [22]							A globally coordinated establishment of a framework of governance, surveillance and reporting of AMR to deal with AMR during and after COVID-19.
Monnet and Harbarth, 2020 [11]				Compliance with IPC measures is essential for controlling the spread of COVID-19 infections and AMR bacteria, as well.			
Heydargoy, 2020 [20]			Expand virtual consultations systems with reduced cost to decrease antibiotic use in self-medication practices in people who cannot leave home because of the pandemic.				
Zhu et al., 2021 [13]						Monitoring of consultations, antibiotics prescribing and AMR should continue during and beyond the COVID-19 pandemic to determine the long-term impact on prescribing behaviour among clinicians.	
Ashiru-Oredope et al., 2021 [8]				Increased awareness of antimicrobial guidelines and improvements on infection prevention and control.		Technology as a facilitator for AMS activities. Better use of technology (virtual platforms and remote working).	
